# Assessment of the knowledge, attitudes and practices of Lebanese shoppers towards food labeling: The first steps in the Nutri-score roadmap

**DOI:** 10.12688/f1000research.75703.1

**Published:** 2022-01-24

**Authors:** Maha Hoteit, Nour Yazbeck, Ayoub Al-Jawaldeh, Cecile Obeid, Heba Abdul Fattah, Marwa Ghader, Hala Mohsen

**Affiliations:** 1 Faculty of Public Health, Lebanese University, Beirut, 961, Lebanon; 2 PHENOL research group (Public HEalth Nutrition prOgram-Lebanon), Faculty of Public Health, Lebanese University, Beirut, 961, Lebanon; 3 Lebanese University Nutrition Surveillance Center (LUNSC), Lebanese Food Drugs and Chemical Administrations, Lebanese University, Beirut, Lebanon; 4 World Health Organization Regional Office for the Eastern Mediterranean, World Health Organization, Cairo, 11371, Egypt; 5 Faculty of Nursing and Health Sciences, Notre Dame University, Zouk Mosbeh, Lebanon

**Keywords:** Food labels, knowledge, attitude, practices, Lebanon

## Abstract

**Background**: Food labeling is a fundamental educational tool for advocating for public awareness. It emphasizes knowledge of the nutrient content of food and thus directs the choice towards the healthiest food products.

This cross-sectional survey aimed to assess the knowledge, attitudes, and practices (KAP) regarding nutrition label use in Lebanon through a valid questionnaire.

**Methods:** Overall, 768 participants (mean age: 30.8 ±12, males: 60.2%) were recruited randomly between February and May 2020. We used word of mouth and social media to recruit our sample population.

**Results**: Social media was the most accessed tool to attain nutrition information by responders (39.8%). More than half the participants expressed positive attitudes to check information related to sugars (66.4%), vitamins (64.9%), total fats (61.7%), proteins (59.1%), and calories (58.7%) on the food label. Expiry date, price, and brand name were the top three considerations while reading food labels. About half (46.5%) reported to “always” look at the food label. Responders reported reading labels related primarily to sugars (44.3%), calories (38.8%), and total fats (36.8%). The optimal total KAP score was 46; our findings revealed a mean KAP score of 14.46 ±7 (31.4%). When categorizing the KAP scores, 15% had high scores, and 85% scored low. Spearman’s coefficients showed positive correlations between knowledge-attitude, knowledge-practice, and attitude-practice scores, with p<0.001. The regression analysis revealed that gender, age, BMI, residency area, educational level, university degree, health and diet statuses, and activity level were significant predictors of the KAP score. Being on a diet had the highest odds (OR=3.107, CI=1.904-5.072, p<0.001).

**Conclusion:** The low awareness of food labels leads Lebanese people to choose unhealthy food options. A planned educational program is recommended to ease the interpretation of these labels.

## Introduction

Non-communicable diseases (NCDs) account for over 35 million disease cases per year and two-thirds of the world's deaths. ^1^ Nutrition-related non-communicable diseases (N-NCDs), mainly diabetes, cancer, hypertension, and cardiovascular diseases, are highly prevalent in most countries in the Eastern Mediterranean Region (EMR). ^2^ In Lebanon, it is estimated that NCDs accounted for 91% of all deaths in 2016. ^3^ Thus, as a preventive action plan, the Lebanese Ministry of Public Health (MOPH) developed a national NCD prevention and control plan (NCD-PCP), that is yet to be implemented. ^4^ Dietary guidelines and labeling legislations are considered an effective tool for creating a healthy food environment to reduce the global burden of NCDs. ^5^ Moreover, the nutrition facts provided on food labels could drive favorable consumers’ behaviors. ^6^ In the United States of America (US), 98% of FDA-regulated packaged foods have nutrition facts panels (NFPs). ^7^ As for Europe, 84% of products have nutrition labels. ^8^ In Lebanon, nutrition labeling is regulated by mandatory standards: NL 206, NL 719 which address the labeling requirements for foods. ^9^ Food labeling is required for most prepared foods such as breads, cereals, canned and frozen foods, snacks, desserts, drinks, among others. Ingredients list, nutrition facts, food allergen declarations and date marking must be available as well. ^9^ The use of food label information is influenced by multiple elements, including comprehension difficulties, promotions, price, educational level, attention, and memorizing the information to apply it to a consequent food-related decision. ^10^ Thus, a combination of these factors may propel the consumer to prefer one product over another. ^10^ However, the presence of detailed nutrition information on the food package does not necessarily guarantee a healthy choice. ^6^ For example, it was found that most consumers (78%) could notice nutrient differences between food products; however, fewer (20%) were able to calculate the contribution of food nutrients to the daily intake. ^10^ Additionally, a recent cross-sectional study revealed that the Health Star Rating, a front-of-pack labelling system that rates the overall nutrition profile of packaged food and assigns it a rating from 0.5 to 5 stars, resulted in a greater willingness to pay for healthier products. ^6^ The cognitive processing model considers decision-making as a high-level cognitive process defining how people think, perceive, remember and learn. ^10^ In other words, it represents the acquisition and storing of knowledge related to any topic leading to a corresponding behavior. ^10^ As for nutrition labels, this model showed that food purchasing behaviors depend heavily on having prior knowledge via three interlinked pathways. ^10^ Prior knowledge could enable consumers to disregard marketing attributes. ^10^ Besides, it facilitates the comprehension and memory of nutrition information. ^10^ As a result, the stored information supports the choice of healthy food options. ^10^ The model concluded that consumers who are more knowledgeable about food nutrients are more likely to develop positive attitudes and use label information productively and correctly. ^10^ A previous study reported that two-third of the consumers with a particular interest in healthy eating actually payed attention to food labels when shopping. ^11^ This result suggests that across most countries, the effectiveness of food labelling varies with culture, nutritional knowledge and demographic characteristics of the population. ^11^ Thus, it is a high priority to point out the factors affecting Lebanese consumers’ interpretation of food labels, to formulate new regulations or update existing ones. The aim of this study was to assess consumers’ knowledge, attitudes and practices (KAP) regarding the information on food labels, and to investigate the correlates of low levels of KAP among Lebanese shoppers.

## Methods

### Study design and data collection

This was a cross-sectional study conducted between February and May 2020. The estimated minimum sample size was 384 (as per the Epi-info statistical software developed by the Center for Disease Control and Prevention Version 7.2,), and we eventually recruited 768 participants from the different Lebanese governorates. Lebanese participants aged 15 to 64 years old were eligible to participate in the study. Non-Lebanese individuals and those who did not fit our age recommendations were disregarded while collecting data. Due to the national lockdown imposed due to the COVID-19 pandemic, a self-constructed and validated questionnaire was filled online. We used word of mouth and social media to recruit our population. We gave a brief explanation of our valuable research objectives for each responder. The collected data were solely used for scientific and research purposes.

### Questionnaire

A 41-item questionnaire was adapted after conducting an extensive review of the literature and based on instruments used in previous studies. ^12^^–^^14^ It was comprised of four parts: the first collected information on demographic, socio-economic and health-related data; the second part (11 questions) focused on the knowledge related to nutrition labels; the third (five questions) addressed the attitudes, and the fourth part (12 questions) investigated the responder’s practices. The questionnaire was drafted in English, subsequently translated into Arabic, the native language of the participants. It was filled online, and it took the participant around 20 minutes to fill it. The completion of the questionnaire was voluntary and anonymous. Firstly, our formulated questionnaire was piloted with 200 respondents to check its acceptability. Its internal consistency and validity were assessed using Cronbach’s alpha (α = 0.7); observed alpha values were high for all the questionnaire sections. As the pilot study justified its validity and reproducibility, the questionnaire was then employed for further data collection. The findings from the pilot study were not considered in the final analysis.

### Scoring criteria of the knowledge, attitudes, and practices

In regard to knowledge, a score of 1 point was granted for responders who answered correctly. Otherwise, those who reported a wrong answer, or failed to give any response (I don’t know response) were given no mark. For each respondent, an overall knowledge score was calculated, by adding the scores from all responses. The respondent could earn a maximum score of 14. The mean knowledge score for our sampled population was then calculated. Regarding the attitudes, and in a similar concept, one point was allocated for each appropriate response; assumed to be a positive attitude. However, a score of zero was issued for undesirable responses which were considered as negative attitudes. For each respondent, an overall attitude score was calculated, by adding the scores from all responses, with a maximum score of 16. The mean attitude score for the overall population was then derived. Likewise, the practice score was computed by adding the respondent's number of appropriate responses over a score of 16. The relevant practice warranted one score for the respondent, whereas an improper practice left the respondent with no score granted. Mean practice score for each respondent was calculated by dividing the total practice score by 16. The resulting scores for the KAP were summed up to generate individualized KAP scores. The respondents could get a maximum KAP score of 46. The KAP score, therefore, was categorized into two levels. A low KAP (<23) and a high KAP (≥23). It is worth noting that the evaluation of the appropriateness of the responders’ KAPs was literature-based, with no subjective judgment.

### Statistical analysis

Data were analyzed using the Statistical Package for Social Sciences (SPSS) software version 25. Demographic, socio-economic, and health-related conditions and the responses related to the knowledge, attitudes, and practices were analyzed descriptively. Data were represented as means ± standard deviation (SD) for continuous variables, and as frequencies (N) and percentages (%) for the categorical ones. The Shapiro-Wilk test indicated a non-normal distribution for the KAP scores, and thus, we used the Mann-Whitney U and Kruskal-Wallis tests to assess differences in the mean KAP scores. The Mann–Whitney U test is suitable for the independent variables with two groups (such as gender), while the Kruskal–Wallis test was used for independent variables including more than two groups (residency, for instance). We also referred to the Spearman’s rank correlation coefficient (rho) to examine the associations between KAP scores. A binary logistic regression was conducted to determine the predictors of KAP scores. A confidence interval of 95% was applied, and the level of significance was determined at 5%.

### Ethical considerations

This study received approval from the ethical committee of the Lebanese University (protocol code CUER # 22-2020). The study’s design and analyses were conducted according to the guidelines of the Declaration of Helsinki. Adult respondents and minors’ families provided a written informed consent before filling the questionnaire, and their confidentiality was protected. We received written informed consent from the adult respondents and minors’ family for the publication of this data.

## Results

### Demographic and socio-economic characteristics

A total of 768 individuals were included in our analysis. The mean age of the overall sample was 30.8 ± 12 years. Adults (25-64 years old) represented 57.8% of the total population, while the remaining were youth (15-24 years old). Male participants constituted the dominant proportion (60.2%). More than half (59.4%) were single or divorced, while 40.6% were married. Moreover, the majority (61.3%) had no children. 49.5% of respondents were working, 35% had no job, 14.3% were housewives, and 1.2% were retired. As for the monthly wage, about half the participants (49.6%) had no salary at all, and another salient proportion (31.9%) were paid less than 1000$ a month. However, 17.3% had a salary of 1000$-3000$, and just 1.2% were earning more than 3000$ per month. Regarding their educational levels, 53.9% were university graduates or still studying at university, among which 76% had Bachelor’s degrees. Besides, 13.7% were studying at general secondary schools, 11.6% were studying at technical secondary schools, 11.2% were studying at elementary/intermediate schools, whereas 9.6% were not attending school at all. Among the final sample, around half the population (49.8%) were living in Beirut and the Mount Lebanon area. The remaining were living in South Lebanon (19%), and North Lebanon (31.2%) areas. Moreover, 45.5% of people in this study had normal BMI values, and 30.5% were overweight. Obesity and overweight status were more prevalent among the male participants (p<0.001). These findings are presented in Table 1.

**Table 1.** Demographic and socio-economic characteristics of the study participants.

Variable	Category	Total		Females		Males		P-value
		N	%	N	%	N	%	
Age categories	Youth (15-24)	324	42.2	203	43.9	121	39.5	0.233
	Adults (25-64)	444	57.8	259	56.1	185	60.5	
BMI categories	Underweight	32	4.2	26	5.6	6	2	< 0.001
	Normal	350	45.5	242	52.5	106	34.8	
	Overweight	234	30.5	121	26.2	113	37	
	Obese	152	19.8	72	15.6	80	26.2	
Gender	Male	462	60.2	NA	NA	NA	NA	NA
	Female	306	39.8	NA	NA	NA	NA	
Residency	Beirut	78	10.2	40	8.6	38	12.4	< 0.001
	South Lebanon	146	19	83	18	63	20.6	
	North Lebanon	240	31.2	140	30.3	100	32.7	
	Mount Lebanon	304	39.6	199	43.1	105	34.3	
Marital status	Married	312	40.6	195	42.2	117	38.2	0.294
	Not Married	456	59.4	267	57.8	189	61.8	
Having children	No	471	61.3	277	60	194	63.4	0.364
	Yes	297	38.7	185	40	112	36.6	
	Unemployed	269	35	163	35.3	106	34.6	
	Retired	9	1.2	0	0	9	2.9	
	Housewife	110	14.3	110	23.8	0	0	
	Employed	380	49.5	189	40.9	191	62.4	
Employment profession	Medical	32	8.4	25	13.2	7	3.6	<0.001
	Non-Medical	348	91.6	164	86.7	184	96.3	
Monthly wage	<1,000$	245	31.9	144	31.2	101	33	<0.001
	1,000$ - 2,000$	111	14.4	39	8.4	72	23.5	
	2,000$ - 3,000$	22	2.9	7	1.5	15	4.9	
	>3000$	9	1.2	0	0	9	2.9	
	No salary	381	49.6	272	58.9	109	35.6	
Educational level	Did not attend school	74	9.6	41	8.9	331	10.8	<0.001
	Elementary/Intermediate Schools	86	11.2	45	9.7	41	13.4	
	General Secondary School	105	13.7	62	13.4	43	14.1	
	Technical Secondary School	89	11.6	50	10.8	39	12.7	
	University	414	53.9	264	57.10	150	49	
University degree	Bachelors’	315	76	189	71.5	126	84	<0.001
	Masters’	91	22	72	27.2	19	12.6	
	Ph.D.	8	2	3	1.4	5	3.3	
University major	Medicine/pharmacy	19	4.6	10	3.7	9	5.9	< 0.001
	Business	88	21.3	36	13.6	52	34.4	
	Engineering	37	8.9	14	5.3	23	15.2	
	Agriculture	1	0.2	1	0.4	0	0	
	Public health	66	16	59	22.3	7	4.6	
	Literature	61	14.7	50	18.8	11	7.3	
	Tourism	10	2.4	5	1.9	5	3.3	
	Education	11	2.7	10	3.8	1	0.6	
	Law	32	7.7	20	7.5	12	8	
	Arts	29	7	19	7.2	10	6.61	
	Sciences	58	14	39	14.7	119	12.6	
	Religion	2	0.5	1	0.4	1	0.6	

### Health and lifestyle conditions

As regards their health status, an appreciable proportion (42.7%) had medical conditions. Specifically, 22.9% had nutrition-related chronic diseases (diabetes, hypertension, cardiovascular and kidney disorders); 22.5% had gastrointestinal disorders (including gastroesophageal reflux disease (GERD), chronic constipation, and intolerances); 13.4% had thyroid disorders, and 3.2% were anemic. The remaining (38%) reported other health conditions (neurological, depression, polycystic ovarian syndrome, among others; Table 2). About one quarter of our sampled population were physically active (attending gym), active males (31.7%) were significantly higher than active females (20.8%), p=0.001. Additionally, 34.2% admitted that they were restricted to specific diets. It was shown that the most prevalent diets were weight loss (55.2%), low carbohydrate (15.2%), and weight gain (8.4%). Others were following intermittent fasting (6.6%), therapeutic (4.9%), low-fat (4.5%), vegan/vegetarian (4.5%), and gluten-free (0.4%) diets. More than half of the female dieters (61.4%) were following a weight loss diet, which was higher than that of males by 18.6%, p<0.001 ( Table 2).

**Table 2.** Health and lifestyle conditions of the study participants.

Variable	Category	Total		Females		Males		P-value
		N	%	N	%	N	%	
Medical conditions	Yes	328	42.7	224	48.52	216	70.6	<0.001
	No	440	57.3	238	51.5	90	29.4	
Type of medical condition	Nutrition-related (diabetes, cardiovascular diseases, hypertension, Kidney disorders)	1432	22.9	82	17.6	61	40.5	<0.001
	Gastro-intestinal disorders	143	22.5	101	21.8	422	27.9	
	Thyroid gland disorders	861	13.4	74	16	12	8	
	Anemia	20	3.2	20	4.3	0	0	
	Others	243	38	178	36.2	2242	3	
Physical activity	No	575	74.9	366	79.2	209	68.3	0.001
	Yes	193	25.1	96	20.8	97	31.7	
Diet	No	505	65.8	286	61.9	219	71.6	0.007
	Yes	263	34.2	176	38.1	87	28.4	
Diet type	Weight loss	124	55.2	91	61.4	33	42.8	<0.001
	Weight gain	19	8.4	7	4.7	12	15.6	
	Low carbohydrates	39	15.2	16	18.9	23	29.9	
	Low fat	8	4.5	4	2.7	4	5.1	
	Vegan/vegetarian	8	4.5	7	4.7	1	1.3	
	Therapeutic	11	4.9	10	9.4	1	1.3	
	Intermittent fasting	15	6.6	12	2.6	3	3.9	
	Gluten-free	1	0.4	1	0.7	0	0	

### Knowledge related to the sources of nutritional information and reading of nutrition labels

**Figure 1.**
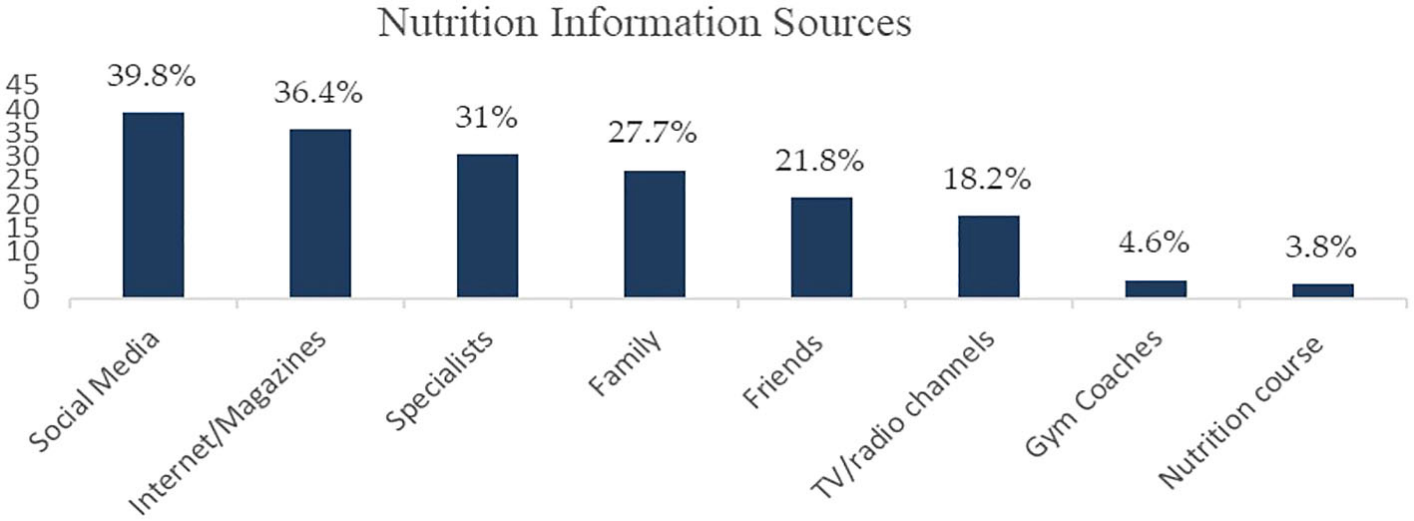
Sources of nutrition information (% of participants).

Nutrition information may be accessed from varied sources, and therefore, we asked our participants to report their frequently used sources to obtain nutrition-related information. Our findings showed that social media platforms were used more frequently (39.8%) ( Figure 1). Almost 36.4% of Lebanese shoppers relied on Internet and magazines as data sources, based on our survey ( Figure 1). On the other hand, a salient proportion of our sampled population (31%) accessed accurate nutrition facts from specialists, such as physicians and dietitians ( Figure 1). Family (27.7%), friends (21.8%), and TV/radio channels (18.2%) were also substantial information sources. Otherwise, the minority reported referring to gym coaches (4.6%) or taking nutrition courses (3.8%) ( Figure 1).

Participants’ responses to knowledge questions are described in Table 3. For knowledge scoring, participants were asked to calculate the number of calories contained in 325 grams of condensed milk (calories per 100 grams were given). They also had to choose the order in which food ingredients appeared on the front of the package. Their familiarity with the “sugar-free” claims indication was also assessed. Additionally, we assessed their awareness of common food additives’ function (xylitol, sorbitol, and aspartame), and whether these sweeteners could have a laxative effect or not. Furthermore, they had to decide if monosodium glutamate (MSG) can be consumed by hypertensive patients and if hydrogenated oils were healthy or unhealthy food ingredients. Moreover, we examined their familiarity with six nutrition symbols: “vegetarian”; “non-vegetarian”; “vegan”; “gluten-free”; “trans-fat-free”; and “genetically modified organisms (GMO)” symbols. The final assessed knowledge area focused on E-numbers and their corresponding functions when added to food.

**Table 3.** Knowledge, attitudes, and practices of study participants (descriptive analysis).

	Appropriate answer	Positive answer* N (%)	Negative answer* N (%)	Don’t know N (%)
**Knowledge**				
Calculation of calories present in 395 g of “Nestle” can	1283 Kcal	196 (25.6)	49 (6.4)	522 (68)
The order of the ingredients listed on the food package	Descending	221 (28.8)	155 (20.2)	392 (50.6)
“Sugar Free” claim indication	No sugar, contains sweetener	378 (48.6)	265 (34.9)	130 (16.5)
Function of Xylitol, Sorbitol, and Aspartame	Sweeteners	174 (22.7)	13 (1.7)	581 (75.6)
Laxative effect of sweeteners	Laxative	310 (40.4)	26 (3.4)	432 (56.2)
Monosodium glutamate (MSG) for hypertensive patients	Not allowed	48 (6.3)	4 (0.5)	716 (93.2)
Healthfulness of hydrogenated oils	Not healthy	322 (42)	66 (8.6)	379 (49.4)
Guess of the “Vegetarian” symbol indication (Green dotted)	Suitable for vegetarians	18 (2.3)	13 (1.7)	737 (96)
Guess of the "Non-Vegetarian" symbol indication (Red dotted)	Not suitable for vegetarians	8 (1)	20 (2.6)	740 (96.6)
Guess of the “Vegan” symbol indication	Suitable for vegans	32 (4.2)	132 (14.9)	604 (78.9)
Guess of the “Gluten-Free” symbol indication	Suitable for celiac disease patients	115 (15)	18 (1)	632 (84)
Guess the “Trans-Fat” symbol indication	Contains no trans-fat	7 (0.9)	68 (8.9)	692 (90.2)
Guess the “GMO” symbol indication	Genetically modified product	10 (1.3)	45 (5.9)	713 (92.8)
Familiarity with the E-labels and does each E ranges	Know what the “E”s are and their functions	56 (7.3)	29 (3.8)	683 (88.9)
**Attitudes**				
The nutrition facts panel (NFP) beneficial use	Beneficial	557 (72.5)	211 ( 27.5)	-
Mandating of Nutrition Labels	Necessary	687 (89.5)	36 (4.7)	45 (5.8)
The interest of looking over “calories” on the NFP	Interested	451 (58.7)	162 (21.1)	155 (20.2)
The interest of looking over “carbohydrates” on the NFP	Interested	343 (44.7)	167 (21.7)	258 (33.6)
The interest of looking over “total fats” on the NFP	Interested	474 (61.7)	212 (27.6)	82 (10.7)
The interest of looking over “proteins” on the NFP	Interested	454 (59.1)	206 (26.8)	108 (14.1)
The interest of looking over “sugars” on the NFP	Interested	510 (66.4)	246 (32)	12 (1.6)
The interest of looking over “fibers” on the NFP	Interested	358 (46.6)	136 (17.7)	274 (35.7)
The interest of looking over “saturated fat” on the NFP	Interested	129 (16.8)	32 (4.2)	607 (79)
The interest of looking over “trans-fat” on the NFP	Interested	96 (12.5)	17 (2.2)	655 (85.3)
The interest of looking over “cholesterol” on the NFP	Interested	363 (47.3)	151 (19.7)	254 (33.1)
The interest of looking over “MUFA” on the NFP	Interested	43 (5.6)	13 ( 1.7)	712 (92.7)
The interest of looking over “PUFA” on the NFP	Interested	37 (4.8)	11 (1.4)	720 (93.8)
The interest of looking over “sodium” on the NFP	Interested	301 (39.2)	146 (19)	321 (41.8)
The interest of looking over “vitamins” on the NFP	Interested	498 (64.9)	191 (24.9)	78 (10.2)
The interest of looking over “minerals” on the NFP	Interested	340 (44.3)	181 (23.6)	247 (32.2)
**Practices**				
Frequency of looking over nutrition labels	Always	357 (46.5)	411 (53.5)	-
Comparison between two food products based on the nutrition facts NFP panel of each	Compare	271 (35.5)	497 (64.7)	-
Looking over “calories”	Yes	298 (38.9)	315 (41)	155 (20.2)
Looking over “carbohydrates”	Yes	204 (26.6)	306 (39.8)	258 (33.6)
Looking over “total fats”	Yes	283 (36.8)	403 (52.2)	82 (10.7)
Looking over “proteins”	Yes	257 (33.5)	403 (52.5)	108 (14.1)
Looking over “saturated fats”	Yes	88 (11.5)	73 (9.5)	607 (79)
Looking over “trans-fat”	Yes	60 (7.8)	53 (6.9)	655 (85.3)
Looking over “cholesterol”	Yes	203 (26.4)	311 (40.5)	254 (33.1)
Looking over “MUFA”	Yes	23 (3)	33 (4.3)	712 (92.7)
Looking over “PUFA”	Yes	22 (2.9)	26 (3.4)	720 (93.8)
Looking over “sugars”	Yes	340 (44.3)	416 (54.2)	12 (1.6)
Looking over “fibers”	Yes	192 (25)	302 (39.3)	274 (35.7)
Looking over “vitamins”	Yes	252 (32.9)	437 (57)	78 (10.2)
Looking over “minerals”	Yes	165 (21.5)	356 (46.4)	247 (32.7)
Looking over “sodium”	Yes	159 (20.7)	288 (37.5)	321 (41.8)

Accordingly, the understanding of nutrition labels was rated by grading an overall knowledge score for our study population, with a maximum possible score of 14. The mean ± SD knowledge score was 2.46 ± 1.93 (17.6%) for the overall population, 2.68 ± 2.07 for females, and 2.13 ± 2.68 for males, p=0.001 ( Table 4).

**Table 4.** Mean scores for the knowledge, attitudes, and practices (KAP) and the mean KAP for the overall population and by gender.

	Range of score	Overall mean ± SD	Overall mean (%) ^(b)^	Females mean ± SD	Males mean ± SD	P-value
Knowledge score	0-14	2.46 ± 1.935	17.6%	2.68 ± 2.071	2.13 ± 1.658	0.001
Attitudes score	0-16	6.24 ± 2.778	39%	6.39 ± 2.834	6.01± 2.680	0.072
Practices score	0-16	5.76 ± 3.787	36%	6.35 ± 3.982	4.89 ± 3.282	<0.001
Total KAP score ^(a)^	0-46	14.46 ± 7	31.4%	15 ± 7	13 ± 6	<0.001

^(a)^ Total KAP score is the sum of the knowledge, the attitude and the practice score.

^(b)^ Overall mean (%) is the product of the overall mean by hundred divided by the upper limit of each score, e.g., knowledge overall mean (%) = (2.46*100)/14= 17.6%.

### Attitudes towards nutrition labels

Participants’ responses to attitude questions are described in Table 3. We observed how our participants do perceive the beneficial use of the nutrition labels and their mandating. Also, we inquired about participants’ interests in looking over information related to calorie’s, macronutrients, and micronutrients on the nutrition facts panel. Study findings showed that the attitude of Lebanese shoppers towards nutrition facts in food product were mostly to check information related to sugars (66.4%), vitamins (64.9%), total fats (61.7%), proteins (59.1%), calories (58.7%), cholesterol (47.3%), fibers (46.6%), carbohydrates (44.7%) minerals (44.3%) among which most importantly sodium (39.2%). In contrast, they gave less consideration for information related to saturated fats (16.8%), trans-fat (12.5%), monounsaturated fatty acids (MUFA) (5.6%), and polyunsaturated fatty acids (PUFA) (4.8%).

Therefore, by responding to these questions, participants can optimally get an attitude score of 16. The mean ± SD attitude score was 6.24 ± 2.78 (39%) for the overall population, 6.39 ± 2.83 for females, and 6.01 ± 2.68 for males, p=0.072 ( Table 4).

### Practices of nutrition labels use

**Figure 2.**
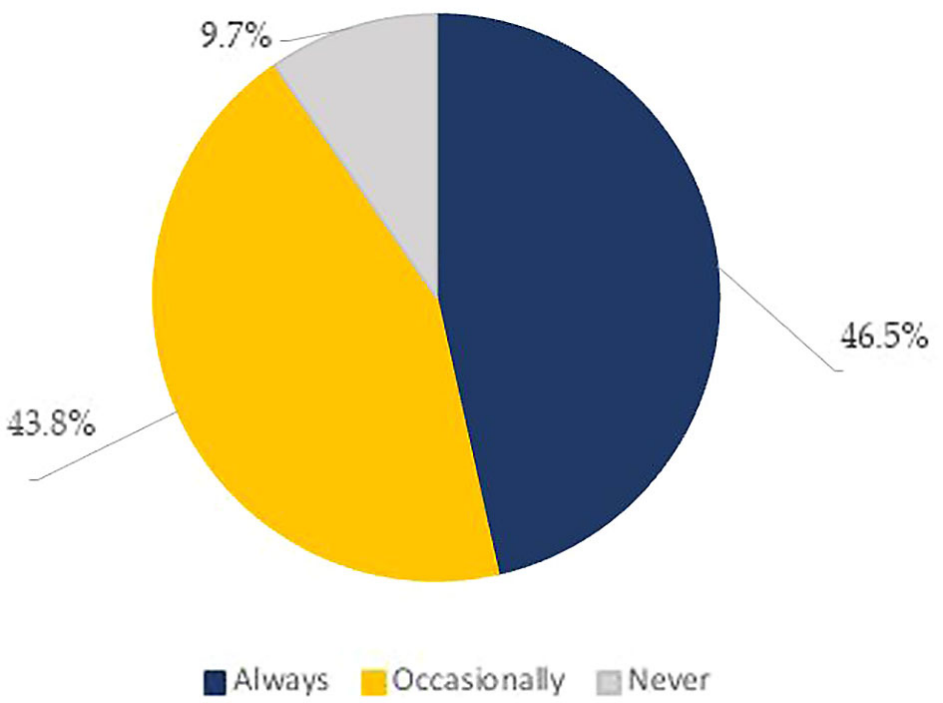
Frequency of looking at Food labels (% of participants).

In practice, the top three information searched for when looking at the nutrition facts panel were: expiry date (75.2%), price (60.6%), and brand name (50.8%) ( Table 5). Whereas the least sought for information was: nutrition and health claims (24.8%), nutrition content (24.3%), food weight (13.6%), and presence of preparation and cooking instructions (9.4%) ( Table 5). Practice scores were based on 16 questions, including the frequency of checking nutrition labels, the act of comparing the nutrient content of food products, and looking at calories, macronutrients, and micronutrients on the food label. As for the frequency of checking the food label, about half of our sample population (46.5%) reported to “always” read the food label, whereas 43.8% of the respondents reported reading them occasionally and only 9.7% admitted not to read it at all ( Figure 2, Table 3). Besides this, when hesitating between two food products, the majority (64.7%) reported that they don’t refer to the NFP to base their choices ( Table 3).

**Table 5.** Considerations while reading food label.

Considerations while reading food labels	N	%
Expiry date	578	75.2
Price	466	60.6
Brand name	390	50.8
Ingredients	267	47
Nutrition content	187	24.3
Country of origin	257	33.5
Food weight	105	13.6
Presence of preparation recipe	72	9.4
Presence of nutrition and health claims	191	24.8

In contrast to their attitudes towards the most searched information when looking at the NFP, in practice, Lebanese shoppers looked for the following nutrient information: sugars (44.3%), followed by calories (38.8%), total fats (36.8%), proteins (33.5%), and vitamins (32.9%). Others read labels related to saturated fats (11.5%), carbohydrates (26.6%), cholesterol (26.4%), fibers (25%), minerals (21.5%), sodium (20.7%), and trans-fats (7.8%). A minority were concerned to check information about monounsaturated fatty acids (MUFA) (3%), and polyunsaturated fatty acids (PUFA) (2.9%) ( Table 3).

Respectively, by responding to these questions, participants could get a maximum practice score of 16. The mean ± SD practice score was 5.76 ± 3.78 (36%) for the overall population, 6.35 ± 3.982 for females, and 4.89 ± 3.28 for males, p<0.001 ( Table 4).

### Total KAP scores

The obtained knowledge, attitudes, and practices scores were summed up to determine the total KAP score. Subsequently, the respondent could get a maximum total KAP score of 46. Our findings showed a total KAP score with a mean ± SD of 14.46 ± 7 (31.4%) for the overall population, 15 ± 7 for females, and 13 ± 6 for males, p<0.001 ( Table 4).

### Correlations between knowledge, attitudes, and practices scores

The Spearman’s coefficients (rho) are presented in Table 6. There was a positive correlation between knowledge and attitude scores (rho = 0.356, p<0.001). Similarly, the knowledge and practice scores were positively correlated (rho=0.38, p<0.001). Correlation findings revealed a strong positive association between attitudes and practices with a Spearman’s coefficient rho= 0.562, p<0.001. These findings demonstrate that knowledge, attitude, and practice scores increased simultaneously, and these scores were significantly correlated.

**Table 6.** Correlations between knowledge, attitudes, and practices scores: Spearman’s coefficients.

	Correlation coefficients (rho)	P-value
Knowledge-attitude scores	0.356	<0.001
Knowledge-practice scores	0.380	<0.001
Attitude-practice scores	0.562	<0.001

### Association of demographic, socioeconomic and health-related conditions with the KAP score based on univariate analysis

The KAP score was significantly lower for men (13 ± 6) than women (15 ± 7), p<0.001. Adults had higher scores when compared to youth (15 ± 7 vs. 14 ± 7, p=0.419). Although underweight, overweight, and obese participants had similar scores (14 ± 7), those with normal BMI had a higher mean score (15 ± 7), p<0.001. In addition, the KAP score was significantly lower for North Lebanon residents (13 ± 6, p<0.001). Further, medical sector workers had higher mean scores, as opposed to non-medical sector workers (18 ± 7 versus 14 ± 6, p=0.012). Participants earning the highest income (>3000$/month) had the highest mean score (19 ± 8), p=0.008. Non-married (15 ± 7) and childless (15 ± 7) respondents had better mean KAP scores, as opposed to their counterparts, p=0.068, and p=0.074, respectively ( Table 7).

**Table 7.** KAP score mean differences: Mann-Whitney U and Kruskal-Wallis tests.

Variables	KAP score (mean ± SD)	P-value
**Gender**		
Females	15 ± 7	<0.001
Males	13 ± 6	
**Age**		
Adults	15 ± 7	0.419
Youth	14 ± 7	
**BMI**		
Underweight	14 ± 7	<0.001
Normal	15 ± 7	
Overweight	14 ± 7	
Obese	14 ± 7	
**Residency**		
Beirut	17 ± 7	<0.001
North Lebanon	13 ± 6	
South Lebanon	15 ± 7	
Mount Lebanon	14 ± 7	
**Employment profession**		
Medical sector	18 ± 7	0.012
Non-medical sector	14 ± 6	
**Monthly wage**		
<1000$	13 ± 6	0.008
1000-2000$	15 ± 6	
2000-3000$	16 ± 8	
>3000$	19 ± 8	
**Marital Status**		
Married	14 ± 7	0.068
Not-married	15 ± 7	
**Children status**		
Have children	14 ± 7	0.074
Have no children	15 ± 7	
**Education Level**		
Studying at university	16 ± 7	<0.001
Low educational levels	13 ± 6	
**University degree**		
Bachelor	15 ± 7	<0.001
Master	18 ± 9	
Ph.D.	22 ± 7	
**Diet status**		
On diet	17 ± 8	<0.001
Not on diet	13 ± 6	
**Physical activity status**		
Yes	17 ± 7	<0.001
No	14 ± 7	
**Medical conditions**		
Healthy	15 ± 7	0.184
Diseased	14 ± 7	

University students had a higher mean score of 16 ± 7 when compared to lower educational level students, p<0.001. Ph.D. degree holders (22 ± 7) had higher mean KAP scores than those holding Masters’ (18 ± 9) and Bachelors’ degrees (15 ± 7), p<0.001. Those who were restricted to specific diets (17 ± 8) and physically active participants (17 ± 7) had higher mean scores than their counterparts (p<0.001). Healthy respondents had a higher mean KAP score than diseased participants (15 ± 7 versus 14 ± 7, p=0.184; Table 7).

### Predictors of the KAP score: The binary logistic regression

**Figure 3.**
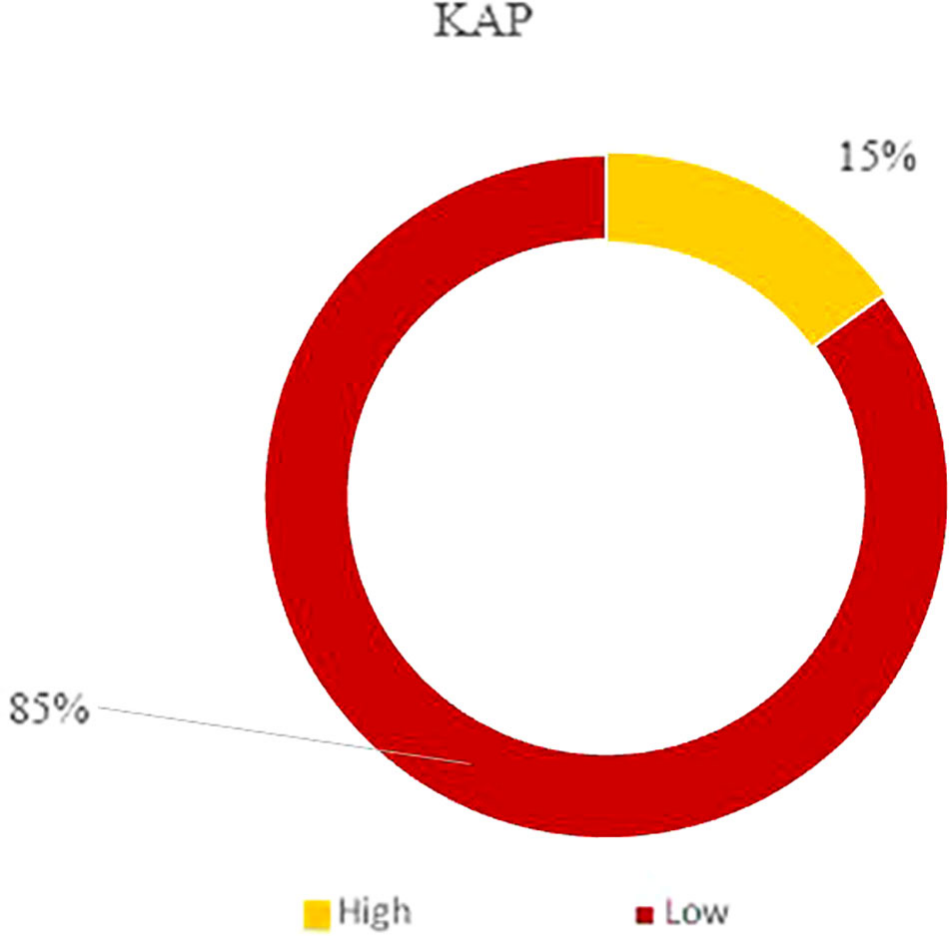
KAP classification.

The KAP score was categorized into two levels: a low KAP (<23) and a high KAP (≥23). The descriptive analysis revealed that 15% of our overall study population had high KAP scores, and 85% scored low for KAP ( Figure 3). Based on these findings, we determined the risk factors contributing to having either a low or a high score by running a binary logistic regression test.

Table 8 shows the binary regression analysis findings, with no adjustment. Males (versus females, OR=0.291, CI=0.159-0.321) were less likely to have a high KAP score by 71%, p<0.001. Adult participants were 1.2 times more likely to have a high KAP score, as opposed to young participants, (OR=1.21, CI=0.727-2.013, p=0.539). In addition, being overweight (OR=1.343, CI=0.349-5.171, versus underweight) and obese (OR=1.322, CI=0.32-5.453, versus underweight) increased the possibility of having a high KAP score by 1.3 times (p=0.668 and p=0.7, respectively). Regarding the residency area, North Lebanon residents had the lowest total KAP scores. In particular, North area residents (versus Beirut residents, OR=0.359, CI=0.161-0.804) were less likely to have high scores by 64.1%, p=0.013.

**Table 8.** Binary logistic regression findings, with no adjustment.

Predictors of the KAP score (0: Low KAP 1: High KAP)	Odds ratio (OR)	95% CI		P-value
		Lower	Higher	
**Gender**				
Female (reference)	1	-	-	-
Male	0.291	0.159	0.321	<0.001
**Age**				
Youth (reference)	1	-	-	-
Adults	1.210	0.727	2.013	0.539
**BMI**				
Underweight (reference)	1	-	-	-
Normal	1.072	0.291	3.949	0.916
Overweight	1.343	0.349	5.171	0.668
Obese	1.322	0.320	5.453	0.700
**Residency**				
Beirut (reference)	1	-	-	-
North Lebanon	0.359	0.161	0.804	0.013
South Lebanon	0.716	0.327	1.569	0.404
Mount Lebanon	0.514	0.252	1.050	0.68
**Studying at University**				
No (reference)	1	-	-	-
Yes	2.040	1.126	3.696	0.019
**University degree**				
Undergraduate (reference)	1	-	-	-
Postgraduate	2.068	1.148	3.725	0.016
**Dieting**				
No	1	-	-	-
Yes	3.107	1.904	5.072	<0.001
**Physical activity status**				
No	1	-	-	-
Yes	2.245	1.364	3.696	0.001
**Medical condition**				
No	1	-	-	-
Yes	0.790	0.480	1.302	0.355

In terms of the educational level, university students (versus lower education level students) were two times more likely to score acceptably (OR=2.04, CI=1.126-3.696), p=0.019. Although studying at university appeared to be a significant predictor, a higher university degree contributed more significantly to the KAP score prediction. Postgraduates had had a two-fold greater probability to have a high KAP score, as opposed to undergraduates (OR=2.068, CI=1.148-3.725), p=0.016.

Interestingly, our findings show that those who were restricted to specific diets (versus non-dieters, OR=3.107, CI=1.904-5.072) and those who were physically active (versus non-actives, OR=2.245, OR=1.364-3.696) were 3 and 2.2 times more likely to score acceptably, respectively (p<0.001). For the final predictor, i.e., the health condition, it was revealed that diseased participants (versus healthy) were less likely to obtain high score by 21% (OR=0.790, CI=0.480-1.302), p=0.355.

## Discussion

This study assessed the KAPs related to the reading of nutrition labels among Lebanese people, using a validated questionnaire. Overall, the mean KAP score was low (14.46 ± 7, 31.4%) which might indicate a lack of public awareness of nutrition label use. In this study, the mean knowledge score was 2.46/14 points (17.6 over 100 points). Social media were the most used platforms (39.8%) to access nutrition information. However, a lower proportion (31%) accessed accurate and reliable nutrition facts from physicians, dietitians, and other specialists. A previous study aiming to assess the relationship between knowledge and the use of nutrition information on food packages reported that Croatian participants had an average nutrition knowledge of 70% (70 over 100 points). ^15^ According to Koen *et al.*, 2018, the knowledge scores of African participants was estimated around 44%. ^16^ As for Italy, a mean nutrition knowledge score of 46% was observed. ^17^ In Arabic countries, a recent observational study by Arfaoui *et al.*, 2021 showed that adult Saudi adult consumers had a total knowledge score of 5.8/13 points (45%), and about 77% of the Saudi participants had an average knowledge score (50th-75th percentiles using the percentile threshold method). ^18^ Between December 2013 and February 2014, a cross-sectional study was conducted among 748 Lebanese shoppers. ^19^ In a previous study, Lebanese shoppers had an average food label knowledge score of 63.1%. ^19^ The massive gap between our obtained knowledge score (17.6%) and that observed in a previous year (63.1%), might be partly related to the study protocol, as we carried out an online survey, which might have affected the comprehension of the questions; however, in the previous study, shoppers were surveyed in supermarkets. Moreover, the latest economic crisis might have driven the interest of Lebanese shoppers into seeking price over quality. In particular, our respondents had low knowledge scores about the E-number additives (E200, E700 …). E-numbers are the chemical names of certain food additives, and they appeared to have a bad reputation among consumers. ^20^ Products containing food additives are usually perceived as unhealthier products. ^20^ A study was conducted to compare E-number labels with “clean” labels, and showed that consumers find “clean” label ingredient lists the safest, healthiest, and the easiest to read. ^20^ Concerning the ability to interpret nutrition symbols on food labels, especially the vegetarian and vegan symbols, only 2.3% and 4.2% (respectively of our sample were able to do it. A study by Berich, H. (2015) done among Kent State University students found that vegetarian dieters were more familiar with such symbols. ^21^ It should be noted that only 1.8% of our participants were on a vegetarian/vegan diet, explaining the unfamiliarity with their corresponding food symbols. The “GMO” symbols corresponding to food containing genetically modified organisms were also unfamiliar for the majority (92.8%) of our respondents. Foods that contain genetically modified organisms was introduced to the US market and appeared on supermarket shelves in 1994. ^22^ Comparing Lebanon to other countries, a cross-cultural survey assessing the knowledge of consumers in the US, Japan, and Italy showed that US consumers were more familiar with GMOs (40.9%) compared with Italian (28.0%) and Japanese (33.3%) consumers. ^22^ Similarly, only 15% of our participants were familiar with the “gluten-free” food symbol. This finding is unsurprising, as only a few of our study population reported following a gluten-free diet. In addition, another important explanation is that we exposed our participants to the “crossed grain gluten-free” symbol, and not to worded gluten-free claims on packages. In Poland, an eye-tracking study of gluten-free cookies showed that consumers were more uncertain about the crossed grain symbol, as compared to verbal statements and gluten-free claims. ^23^ In contrast, about half of our sampled population agreed that “sugar-free”-labeled products are artificially sweetened. However, they had little idea about the artificial additives which act as sugar substitutes: 75.6% of the overall responders were not familiar with the role of xylitol, sorbitol, and aspartame sweeteners. In a different setting, a qualitative study exploring consumer knowledge and understanding of added sugars in the United Kingdom demonstrated that saccharin and aspartame sweeteners were correctly classified by the majority (60% and 80%, respectively) of respondents. ^24^ Sweeteners have been used to increase food flavor and were adopted because they contain few to no calories compared to the high caloric content of sugars. ^25^ Also, an important proportion of our respondents (40.4%) were aware of the laxative effect of these sweeteners. We can assume this is due to having had previous experiences with this side effect after consuming any sugar-free product. As to MSG, the majority (93.2%) of our sample population did not know whether MSG has blood pressure-elevation effects. A nutrition study on Chinese adults observed that MSG intake was associated with a significant increase in blood pressure levels, especially among patients chronically taking antihypertensive medications. ^26^ A previous study aimed to assess the KAP towards the use of MSG in Pakistan explored that 98.3% of the respondents were using MSGs as food flavor enhancers while cooking stews, soups, pottages, sauce, and others (6.6 g/person/week). ^27^ Contrarily to our findings, 42.5% of the Pakistani consumers had high knowledge levels about MSG. ^27^ Regarding hydrogenated vegetable oils, 42% of Lebanese shoppers in our study agreed that they caused detrimental health side effects. In Saudi Arabia, a study was conducted to assess the trans-fat-related knowledge among Saudis; its results showed that around 35.1% of the participants were familiar with the term “hydrogenated oils”, and only 4% classified these ingredients as unhealthy. ^28^ Even though Lebanese shoppers showed high knowledge regarding hydrogenated oils’ health impacts, practice results differed when a cross-sectional survey was conducted on 657 Lebanese adults who completed the US National Institute of Health diet history questionnaire. ^29^ This study found that the mean trans-fatty acids consumption among Lebanese people was double the World Health Organization (WHO) recommendations of 1 percent of total daily energy. ^29^ Partially hydrogenated oils have been found to contain extremely harmful fatty acids, and they cause inflammation and calcification of the arterial cells, increasing the risk of coronary heart disease (CHD). ^30^ When we asked our participants to calculate the energy density of a sweetened condensed milk, the majority either gave a wrong answer or failed to do any calculation (74.4%). Similarly, several studies reported a low understanding of serving size labeling among consumers. ^31^ A study done by Persoskie *et al.*, 2017, showed that Americans could not determine the calorie content of a full ice-cream container. ^32^ Additionally, 21% could not estimate the number of servings equal to 60 g of carbohydrates, 42% could not estimate the effect on the daily calorie intake of one serving, and 41% failed to calculate the percentage daily value (DV) of calories in a single serving. ^32^ The mean attitude and practice scores for our population were 6.24 ± 2.778 (over 16) and 5.76 ± 3.787 (over 16), respectively. Our findings have shown that the knowledge, attitude, and practice scores were positively correlated. It is known that the better the knowledge, the better the attitudes will be, and thus, more appropriate practices will take place. ^33^ Although knowledge itself does not necessarily guarantee a behavior change, it shapes the attitudes towards favorable practices. ^33^ Considering this, our research findings highlight the importance of educating Lebanese shoppers on food labels to ease their interpretation, and this, in turn, may enhance attitudes and practices in the long term. A clear evidence-based front-of-pack label like the nutri-score could be an effective tool to help improve consumers’ diet quality and mitigate the risk of NCDs. ^34^ The Nutri-Score is a front-of-pack label that provides user-friendly information on the nutritional quality of food products. ^34^ It is based on the British Food Standards Agency nutrient profiling system (FSAm-NPS) score. ^34^ The higher the FSAm-NPS score, the lower the nutritional quality of the food. Nutri-score is a rating system that uses five different colours to categorize food products into five groups. ^34^ For example, category A (dark green) suggests higher nutritional quality; however, category E (dark orange) indicates lower nutritional quality. ^34^ The nutri-score is the only front-of-pack nutrition label in Europe having some strong scientific evidence for its effectiveness. ^34^ It has already been adopted by several European countries (Belgium, France, Germany, the Netherlands, Spain, Luxembourg and Switzerland). ^34^ Regarding the observed attitudes, the majority of our participants (72.5%) supported the beneficial use of the NFP. Furthermore, our participants seemed to positively perceive the nutrition labels’ importance, in contrast with a previous cross-sectional study recruiting 748 supermarket Lebanese shoppers in 2014. ^19^ Less than half (44.4%) of the recruited shoppers agreed that reading food labels is very important. ^19^ Besides, our findings are consistent with previous investigations from a cross-sectional study recruiting Iranian students from five different academic majors (including Nutrition, Public Health, Health Services Administration, Paramedical, and Engineering). ^35^ Among 332 students, 89.2% believed that food labels affect nutritional awareness, and 77.4% agreed with their beneficial use. ^35^ Similarly, a US survey aiming to examine the influence of 1990 Nutrition Labeling and Education Act food labels on college students found that 95% of participants perceived the NFP to be useful. ^36^ Moreover, consumers were more concerned about reading nutritional information when they planned to lose weight or follow specific dietary regimens. ^36^ Further, it was mentioned that 81% of participants who read the nutritional panel on product labels were on a weight-loss diet. ^37^ This supports our results, since 55.2% of our sample were dieters, which explains their positive attitudes towards nutrition label use. In addition, our study findings showed that female participants had higher mean attitude scores than males (6.39 ± 2.834 vs. 6.01 ± 2.680, p=0.072). This is because women experience more food-related conflict, and more dissatisfaction with their body weight and shape than men do. ^38^ With regards to nutrition labels mandating, the majority (89.5%) discerned the necessity of such legal actions. In addition to transparency, nutrition facts on food products enable people to determine, choose, and meet their dietary needs. ^39^ The FDA provides effective guidance to the food industry regarding labeling information, depending on the product type. ^39^ In an attempt to assess consumers’ valuation of nutrition labels, data was collected from food shoppers to observe their willingness to pay a premium cost for a box of cookies with a nutritional label. ^40^ Interestingly, that study found that the mean willingness to pay for a box of cookies with a nutritional label was about 11%higher than that of a cookie box without a nutritional label. ^40^ Moreover, expiry date marking (75.2%), price (60.6%), and brand name (50.8%) were the prioritized considerations for of our sample, whereas a smaller proportion admitted considering food ingredients (47%), and nutritional content (24.3%). Similar to our findings, the majority (84.7%) of the shoppers in Tabriz, Iran have been found to look for the expiry and production dates on the food label. ^41^ In addition, 51.6% were looking at food price, and only 8.7% of the participants read food labels to find information about the food additives and artificial colors. ^41^ Further, the date of minimum durability (i.e. best-before or use-by date) was rated the most important piece of mandatory labelling information, with 81% of Irish consumers scoring it as very important. ^42^ Our respondents ranking the food price as a priority consideration was a predictable finding: at the time of our data collection, Lebanon was in a financial crisis, and more than half the population lived below the poverty line. According to the latest data in July 2021, a family's budget for food was around five times the minimum monthly wage. ^43^ In contrast, a survey examining the awareness of food labeling among consumers in groceries in Al-Ain, United Arab Emirates reported that consumer’s responses showed general tendencies for reading the food label (89.5%); however; they read basic information related to production and expiry dates. ^44^ Another study in India showed that the taste, quality, convenience and ease of use were the main reasons for purchasing food among the study participants. ^45^ The majority (81%) looked for the expiry date, and only one third purchased food based on its nutritional value. ^45^ Moving to South Africa (Lesotho), an observational study reported that 40.5% of the participants were interested in reading the nutrition information on food labels, rather than facts related to price, taste, appearance, habit, convenience, or brand name. ^46^ When asked about the frequency at which they read nutrition labels, about half of our responders (46.5%) reported to “always” read the food labels. These findings are considered reasonable when compared to other studies in Arabic countries and worldwide. A research survey showed that only 27.4% of Saudi female college students stated that they always read food labels when purchasing food products. ^47^ In China, however, 59.2% of a survey respondents indicated to “sometimes” look at the label, and only 28.7%” always” read nutrition label. ^48^ Besides, only 21.6% of university Malaysian students reported to “often” use the food label during food purchasing decisions. ^49^ To specify, our responders claimed to read labels relating primarily to sugars (44.3%), followed by calories (38.9%), total fats (36.8%), proteins (33.5%), and vitamins (32.9%). Chinese people had similar reading practices, and were found to look at proteins (51.5%), vitamins (49.8%), and fats (29.4%). ^48^ A preliminary review aimed at assessing the types of label formats that could influence the use of nutritional label among consumers showed that the majority of label users were interested in checking information related to calories, fat, sodium, and cholesterol, and in deciding whether to buy fresh fluid milk. ^50^ Most importantly, the majority (64.7%) of our responders reported that they did not refer to the NFP to choose between two food products. These results are not unusual in Lebanon: only 22.9% of recruited Lebanese shoppers admitted to checking the food labels every time they bought a food product. ^19^ On the other hand, 65% of Ghanaian consumers read food labels before purchase, and 75% based their food selection according to its nutrition content. ^51^ Besides, more than half of Singhalese people recruited in a cross-sectional survey could select the healthiest option with the better food label, when hesitating between two snack options. ^52^ Consistent findings were obtained from our univariate analysis and binary regression analysis regarding the mean KAP score. Our findings revealed that female (15 ± 7), adult (15 ± 7), healthy (15 ± 7), and overweight/obese participants (14 ± 7) were more likely to have a high KAP score. In addition, those with higher educational levels (university level), and those who had completed higher university studies (Master, Ph.D.) had higher KAP scores. Other interesting findings are that those who were restricted to certain diets and the physically active participants had better KAP scores than their counterparts. On the other hand, North Lebanon residents had a lower KAP score (13 ± 6), as opposed to other residency areas. Supporting our findings, a sex-specific analysis of nutrition label use in the US showed that females used nutrition labels more than males (40.7% versus 54.3%, p<0.001). ^53^ Similarly, another study in Malaysia reported that females, adults, tertiary level-educated, and physically active participants had increased odds of nutrition label use. ^54^ Going back to the cognitive processing model, it revealed that some complex tasks related to reading food labels demand high comprehension and interpretation skills. ^10^ Thus, this model explains why a higher educational level was a significant predictor of better KAP in our study findings. ^10^ In addition, knowing more about food labels and consulting them before food purchase can help dieters and those who care about their body shape to pick healthy food options. ^55^ Upon this, having better knowledge about nutrition labels can protect from many NCDs, which explains why our healthy responders had higher KAP scores than diseased participants. This study has identified the KAP scores and the associated factors of KAP related to nutrition label use among the Lebanese public. The findings of this study might advocate for future educational programs clarifying the meaning of crucial nutrition claims and symbols. Although increasing consumers’ awareness is key in leading to better KAP, food manufacturers should also invest in simplifying their nutrition labels presentation to attract more consumers. As discussed before, concerning the nutri-score’s promising application, epidemiological findings among European cohorts found that people who consume more food with higher FSAm-NPS scores (lower nutritional quality) had a higher risk of developing cancers, and a 6% increase in the risk of overall mortality. ^56^ In France, seven out of 10 people check the Nutri-Score, and 84% say they are very likely to pick food products with higher scores. ^57^ Besides, a cross-sectional study recruiting 501,594 adults from ten European countries mentioned that a FSAm-NPS score was calculated for each participant based on the nutritional quality of the food they consume. ^58^ Individuals with a higher score revealed an increased risk of all-cause mortality as well as the incidence of circulatory and gastrointestinal diseases. ^58^

### Limitations and strengths

Our study includes some limitations. Firstly, the cross-sectional design of the survey itself limited the ability to reach causal inference. In addition, the online distribution of the questionnaire in the second period of the study (after COVID-19 pandemic emergence) may pose information and selection biases. Thus, the self-reported data may overestimate the understanding, the positive attitudes, and the appropriate practices regarding nutrition label use. On the other hand, this study has critical strengths. It is the first study in Lebanon adopting a valid and reliable questionnaire to assess the knowledge, attitudes, and practices of Lebanese consumers regarding nutrition label use. Besides, responders were recruited from different areas, had different educational levels, and were of various ages so that the study’s findings could be generalized to the whole population.

## Conclusion

The low awareness of nutrition labels leads Lebanese people to choose unhealthy food options. This study showed an association between the participants’ attitudes, practices and self-reported knowledge. Because nutrition and chronic diseases are interrelated, a planned educational program is recommended to help Lebanese people pick healthy options mindfully. It is necessary to establish educational campaigns about the association between reading nutrition labels and health outcomes. The Food and Drug Administration (FDA) has implemented a “Read the Label” campaign to support children, families, and community leaders in analyzing nutrition labels and to use them effectively. In conclusion, advocating for a nutrition rating system like Nutri-Score in Lebanon is fundamental to mitigate obesity and chronic disease burden. However, one should take into account that nutrition labeling is only one approach to a public health nutrition policy, and it should be complementary with other public health measures and, in particular, nutrition education and communication.

## Data availability

OSF: Assessment of the Knowledge, attitudes and practices (KAP) of Lebanese shoppers towards food labeling: The first steps in the Nutri-score roadmap, https://osf.io/3nh5 ^59^

This project contains the following underlying data:

• Data-Excel-Nutrition Label.xlsx

Data are available under the terms of the Creative Commons Zero “No rights reserved” data waiver (CC0 1.0 Public domain dedication).
