# Supplementary material for: Assessment of the knowledge, attitudes and practices of Lebanese shoppers towards food labeling: The first steps in the Nutri-score roadmap
Source: F1000Res. 2022 May 5;11:84. Originally published 2022 Jan 24. [Version 2] doi: 10.12688/f1000research.75703.2 (PMC9194517; doi:10.12688/f1000research.75703.2)
Supplement: Peer review history

# Peer review: Reviewer response for version 2

**DOI:** 10.5256/f1000research.131276.r136717

**Published:** 2022-06-13

**Authors:** Zahra Esfandiari

**Affiliations:** 1 Food Security Research Center AND Department of Food Science and Technology, School of Nutrition and Food Science, Isfahan University of Medical Sciences, Isfahan, Iran

I confirm that I have studied this submission and approve that all comments are inserted and It is in standard of acceptable scientific manuscript.

Is the work clearly and accurately presented and does it cite the current literature?

Yes

If applicable, is the statistical analysis and its interpretation appropriate?

Yes

Are all the source data underlying the results available to ensure full reproducibility?

Partly

Is the study design appropriate and is the work technically sound?

Partly

Are the conclusions drawn adequately supported by the results?

Yes

Are sufficient details of methods and analysis provided to allow replication by others?

Partly

Reviewer Expertise:

NA

I confirm that I have read this submission and believe that I have an appropriate level of expertise to confirm that it is of an acceptable scientific standard.

# Peer review: Reviewer response for version 2

**DOI:** 10.5256/f1000research.131276.r136718

**Published:** 2022-06-01

**Authors:** Mireille Serhan

**Affiliations:** 1 Department of Nutritional Sciences, Faculty of Health Sciences, University of Balamand, Tripoli, Lebanon

No further comment to make.

Is the work clearly and accurately presented and does it cite the current literature?

Yes

If applicable, is the statistical analysis and its interpretation appropriate?

Yes

Are all the source data underlying the results available to ensure full reproducibility?

Yes

Is the study design appropriate and is the work technically sound?

Partly

Are the conclusions drawn adequately supported by the results?

Yes

Are sufficient details of methods and analysis provided to allow replication by others?

Yes

Reviewer Expertise:

Food safety, Dairy processing, Nutrition and Food Science research, Consumer studies.

I confirm that I have read this submission and believe that I have an appropriate level of expertise to confirm that it is of an acceptable scientific standard.

# Peer review: Reviewer response for version 1

**DOI:** 10.5256/f1000research.79612.r127701

**Published:** 2022-03-29

**Authors:** Mireille Serhan

**Affiliations:** 1 Department of Nutritional Sciences, Faculty of Health Sciences, University of Balamand, Tripoli, Lebanon

The topic is of interest. The study is complete and many important variables are studied to assert a conclusion. Therefore the paper is suitable for indexing:

 With reference to the study design and the questionnaire:

1. Please provide an explanation about why the inclusion criteria for age was set to 15-64 years. 
2. Please demonstrate that the overall number of participants is representative of the population size of participants between 15-64 years. Please demonstrate that the sample size is representative to justify the discussion and conclusion based on the obtained results.
3. It would be better to include rural and urban residence for participants. This is a major factor that would affect the knowledge and attitude of participants, rather than knowing in which governate they are coming from.
4. You mentioned that you used a self-constructed and validated questionnaire that was filled online. And that you used word of mouth and social media to recruit your population. Based on the above, please justify how for the questionnaire that was filled online, how the adult respondents and minors’ families were able to provide a written informed consent before filling the questionnaire. 
5. Please specify in Table 1 that the Numbers in this question are calculated based on the rate of “Sayrafa” during the same day of filling this questionnaire.

Is the work clearly and accurately presented and does it cite the current literature?

Yes

If applicable, is the statistical analysis and its interpretation appropriate?

Yes

Are all the source data underlying the results available to ensure full reproducibility?

Yes

Is the study design appropriate and is the work technically sound?

Partly

Are the conclusions drawn adequately supported by the results?

Yes

Are sufficient details of methods and analysis provided to allow replication by others?

Yes

Reviewer Expertise:

Food safety, Dairy processing, Nutrition and Food Science research, Consumer studies.

I confirm that I have read this submission and believe that I have an appropriate level of expertise to confirm that it is of an acceptable scientific standard.

# Peer review: Reviewer response for version 1

**DOI:** 10.5256/f1000research.79612.r127381

**Published:** 2022-03-21

**Authors:** Zahra Esfandiari

**Affiliations:** 1 Food Security Research Center AND Department of Food Science and Technology, School of Nutrition and Food Science, Isfahan University of Medical Sciences, Isfahan, Iran

The paper: Evaluation of Knowledge, Attitudes and Practices toward the knowledge, attitudes and practices of Lebanese shoppers towards food labeling. It is a good study and a worthy research topic. It is quite relevant to F1000Research topics. However, the article cannot be considered for indexing as it is. It needs a strong revision, as there are serious readability problems and methodological issues that the author(s) must manage effectively. I offer some questions and comments with the hope of helping s/he/them improve their work. I wish the research team the very best of luck as they continue work in this domain.

**Overall evaluation.**

 It is a worthy research topic and an interesting study but the paper needs a strong revision. I hope that the comments are fair and constructive. All Best!

**Introduction:** section needs revision. The author(s) must underline the initial assumptions of the paper and the originality of the paper – contribution to knowledge. I would suggest considering the following five (5) basic elements in the section of introduction as subheadings of Introduction:

 a) Research aim:

 b) Initial assumptions of the paper

 c) Reasoning for the focus of the paper,

 d) Research objectives,

 e) Originality of the paper and contribution to knowledge.

 In the last paragraph of the Introduction, the authors should clearly mention the weaker point of former works (identification of the gaps) and describe the novelties of the current investigation to justify the paper deserves to be indexed.

**Method and material: **This Section needs revision based on the following comments.

1. There is some parts in method and material such as the number of question and scores that needed more explanation; the number of phrases in table for knowledge is 11 but it is mentioned score 14. How is the calculation for knowledge, attitude and practices? It needs more clarification.
2. Authors must explain more about the process of validity and reliability of questionnaire.

**Discussion.**

 Author(s) must explain what is the difference of his study with others and if they are same, why they are similar? Comparison must be with papers after 2013. It needs to expand the importance of the findings. Most of the discussion was overstated and should be revised.

Is the work clearly and accurately presented and does it cite the current literature?

Yes

If applicable, is the statistical analysis and its interpretation appropriate?

Yes

Are all the source data underlying the results available to ensure full reproducibility?

Partly

Is the study design appropriate and is the work technically sound?

Partly

Are the conclusions drawn adequately supported by the results?

Yes

Are sufficient details of methods and analysis provided to allow replication by others?

Partly

Reviewer Expertise:

Food Safety Assessment

I confirm that I have read this submission and believe that I have an appropriate level of expertise to confirm that it is of an acceptable scientific standard, however I have significant reservations, as outlined above.

# Response

**Published:** 2022-04-14

**Authors:** Maha Hoteit

**Affiliations:** Lebanese University, Lebanon

**Reviewer 1**

 The paper: Evaluation of Knowledge, Attitudes and Practices toward the knowledge, attitudes and practices of Lebanese shoppers towards food labeling. It is a good study and a worthy research topic. It is quite relevant to F1000Research topics. However, the article cannot be considered for indexing as it is. It needs a strong revision, as there are serious readability problems and methodological issues that the author(s) must manage effectively. I offer some questions and comments with the hope of helping s/he/them improve their work. I wish the research team the very best of luck as they continue work in this domain.

 Overall evaluation.

 It is a worthy research topic and an interesting study but the paper needs a strong revision. I hope that the comments are fair and constructive. All Best!

 Introduction: section needs revision. The author(s) must underline the initial assumptions of the paper and the originality of the paper – contribution to knowledge. I would suggest considering the following five (5) basic elements in the section of introduction as subheadings of Introduction:

 a) Research aim:

 b) Initial assumptions of the paper

 c) Reasoning for the focus of the paper,

 d) Research objectives,

 e) Originality of the paper and contribution to knowledge.

 In the last paragraph of the Introduction, the authors should clearly mention the weaker point of former works (identification of the gaps) and describe the novelties of the current investigation to justify the paper deserves to be indexed.

**Reply: This is absolutely important and could be more highlighted in the manuscript. We add information about the relevance and the novelty of the current research study in the last paragraph of the Introduction. The study aims and the focus of the paper are clear too.**

**“The usage and understanding of nutrition labels are inadequately registered before in Lebanon, with a current data shortage on this topic.  Thus, it is a high priority to point out the factors affecting Lebanese consumers’ interpretation of food labels, to formulate new regulations or update existing ones. The aim of this study, the first of its kind in Lebanon, was to assess consumers’ knowledge, attitudes and practices (KAP) regarding the information on food labels, and to investigate the correlates of low levels of KAP among Lebanese shoppers. The study findings could serve as an initiative to motivate the national implementation of effective food labeling approaches like the Nutri-score front-of-pack label”.**

 Method and material: This Section needs revision based on the following comments.

 There is some parts in method and material such as the number of question and scores that needed more explanation; the number of phrases in table for knowledge is 11 but it is mentioned score 14. How is the calculation for knowledge, attitude and practices? It needs more clarification.

**Reply: The questionnaire includes 14 knowledge-related questions. However, the knowledge scoring was not based on all of them. For example: The statements inquiring about the “vegetarian”; “non-vegetarian”; “vegan”; “gluten-free”; “Trans-fat”; “GMO” symbols are multiple parts of one question. The same is applied to the attitudes and practices parts of the questionnaire. This is why the total number of the questions and the total scores may differ. We chose the questions which seem reasonable to formulate individuals KAP scores.**

 Authors must explain more about the process of validity and reliability of questionnaire.

**Reply: More details about the validity and reliability are added under the “Questionnaire” subsection of the Methods parts.**

**“Its internal consistency and validity were assessed using Cronbach’s alpha in a preliminary analysis after conducting a pilot study. The observed alpha values were high for all the questionnaire sections (0.909 for knowledge questions, 0.841 for the attitude questions, and 0.836 for the practice questions)”**

 Discussion.

 Author(s) must explain what is the difference of his study with others and if they are same, why they are similar? Comparison must be with papers after 2013. It needs to expand the importance of the findings. Most of the discussion was overstated and should be revised.

**Reply: The discussion section is updated and supported by recent studies by highlighting similarities and differences with other study findings.**

 Reviewer 2: 

 The topic is of interest. The study is complete and many important variables are studied to assert a conclusion. Therefore the paper is suitable for indexing:

 With reference to the study design and the questionnaire:

1. Please provide an explanation about why the inclusion criteria for age was set to 15-64 years. 

**Reply: The youth and adult Lebanese consumers were the target population of the current study. Thus, the age range of 15 to 64 years old was one of the eligibility criteria for our participants.**


**  **

1. Please demonstrate that the overall number of participants is representative of the population size of participants between 15-64 years. Please demonstrate that the sample size is representative to justify the discussion and conclusion based on the obtained results.

**Reply:** **According to the Lebanese Ministry of Public Health (MOPH), the total population aged 15-64 years across all Lebanese governorates in 2020 was 3,079,431. Based on the Epi-Info statistical software, with an acceptable margin of error of 5% and a confidence level of 95%, the minimum representative sample size was 384. Consequently, we recruited 768 participants as a representative sample size in the current study. **

1. It would be better to include rural and urban residence for participants. This is a major factor that would affect the knowledge and attitude of participants, rather than knowing in which governate they are coming from.

**Reply: This is definitely valuable and consistent with the study aims. However, we presented our data after reviewing the literature and in referral to other studies in Lebanon which were often based on the governorates of the participants.**

1. You mentioned that you used a self-constructed and validated questionnaire that was filled online. And that you used word of mouth and social media to recruit your population. Based on the above, please justify how for the questionnaire that was filled online, how the adult respondents and minors’ families were able to provide a written informed consent before filling the questionnaire. 

**Reply: On the first page of the online form of the questionnaire (google form), the participants had to choose if they were consented to participate in the study to continue filling the remaining questions after being informed about study aims, risks, benefits, and confidentiality issues.**


**  **

1. Please specify in Table 1 that the Numbers in this question are calculated based on the rate of “Sayrafa” during the same day of filling this questionnaire.

**Reply: To record their monthly income, participants were asked to determine the exact choice (<1000$; 1000$-2000$; 2000$-3000$;>3000$) according to the rate of “Sayrafa” at the date of investigation. For example, if 1$= 3000 L.L. (Lebanese Lira), a participant with a monthly income of 3,000,000 L.L. had to choose the option “1000$-2000$”.**
